# On Power Idealization Filter Topologies of Lattice Implication Algebras

**DOI:** 10.1155/2014/812145

**Published:** 2014-08-28

**Authors:** Shi-Zhong Bai, Xiu-Yun Wu

**Affiliations:** ^1^School of Mathematics and Computational Science, Xiangtan University, Xiangtan 411105, China; ^2^School of Mathematics and Computational Science, Wuyi University, Jiangmen 529020, China; ^3^Department of Mathematics and Computational Science, Hunan University of Science and Engineering, Yongzhou 425100, China

## Abstract

The aim of this paper is to introduce power idealization filter topologies with respect to filter topologies and power ideals of lattice implication algebras, and to investigate some properties of power idealization filter topological spaces and their quotient spaces.

## 1. Introduction and Preliminaries

By generalizing Boolean algebras and Lukasiewicz implication algebras [1], Xu [2] defined the concept of lattice implication algebra which is regarded as an efficient approach to deal with lattice valued logical systems. Later, Xu and Qin [3] defined the concept of the filer topology of a lattice implication algebra which takes the set of all filters of the lattice implication algebra as a base. Based on these definitions and some results in [4], we introduce and study power idealization topologies with respect to filter topologies and power ideals of lattice implication algebras.

Now we recall some definitions and notions of lattice implication algebras and topological spaces.

Let (*L*, ∧, ∨, 0,1) be a bounded lattice with the greatest 1 and the smallest 0. A system (*L*, ∧, ∨,  ′, →, 0,1) is called a quasi-lattice implication algebra if  ′ : *L* → *L* is an order-reserving involution and →:*L* × *L* → *L* is a map (called an implication operator) satisfying the following conditions for any *x*, *y*, *z* ∈ *L*:

1. *x* → (*y* → *z*) = *y* → (*x* → *z*),
2. *x* → *x* = 1,
3. *x* → *y* = *y*′ → *x*′,
4. *x* → *y* = *y* → *x* = 1 implies *x* = *y*,
5. (*x* → *y*) → *y* = (*y* → *x*) → *x*.

A quasi-lattice implication algebra (*L*, ∧, ∨,  ′, →, 0,1) is called a lattice implication algebra if the implication operator → further fulfils the following conditions:

(6) (*x*∨*y*) → *z* = (*x* → *z*)∧(*y* → *z*),
(7) (*x*∧*y*) → *z* = (*x* → *z*)∨(*y* → *z*).

A lattice implication algebra (*L*, ∧, ∨,  ′, →, 0,1) will be simply denoted by *L*.

Let *L* be a lattice implication algebra and let *φ* be a subset of 2^*L*^. We use *φ* ^*c*^ to denote the complement {*L*∖*A* : *A* ∈ *φ*}, where *L*∖*A* = {*x* ∈ *L* : *x* ∉ *A*}. A subset *τ*⊆2^*L*^ is called a topology on *L*, if *τ* satisfies the following:

1. *∅*, *L* ∈ *τ*,
2. *A*, *B* ∈ *τ* implies *A*∩*B* ∈ *τ*,
3. {*A*_*t*_ ∈ *τ* : *t* ∈ *T*}⊆*τ* implies ∪_*t*∈*T*_ *A*_*t*_ ∈ *τ*.

Elements of *τ* are called *τ*-open sets and the complements of them are called *τ*-closed. The pair (*L*, *τ*) is called a topological space. A subset *B* of *τ* is called a base of *τ*, if for each *A* ∈ *τ* and each *x* ∈ *A*, there exists *B* ∈ *B* such that *x* ∈ *B*⊆*A*.

Let *L* be an implication algebra. A subset *F* of *L* is called a filer, if *F* satisfies the following: (1) 1 ∈ *F*; (2) *x*, *x* → *y* ∈ *F* implies *y* ∈ *F*. The collection of all filters in *L* is denoted by *F*(*L*), or *F* briefly. Clearly, *F* consists a base of some topology *T*_*F*(*L*)_, briefly *T*_*F*_. Usually, *T*_*F*_ is called the filter topology generated by *F*. And the pair (*L*, *T*_*F*_) is called the filter topological space. A subset *U*⊆*L* is called *T*_*F*_-neighborhood of *x* ∈ *L*, or neighborhood of *x* in *T*_*F*_ if *x* ∈ *U* ∈ *T*_*F*_. The set of all *T*_*F*_-neighborhoods of *x* is denoted by *N*_*T*_*F*__(*x*). Since *F*⊆*T*_*F*_ and [*x*) = ∩{*F* : *x* ∈ *F* ∈ *F*} ∈ *F*, [*x*) is the smallest element of *N*_*T*_*F*__(*x*).

The closure operator and interior operator of *T*_*F*_ are denoted by *c* and *i*. Clearly, for every *A*⊆*L*, *c*(*A*) = ∩{*L*∖[*x*) : *x* ∈ *L*, [*x*)∩*A* = *∅*} and *i*(*A*) = ∪{[*x*) : *x* ∈ *L*, [*x*)⊆*A*}. The following proposition describes *c*(*A*).

**Proposition 1 .**

Let (*L*, *T*_*F*_) be the filer topology generated by *F*(*L*). Then for *A*⊆*L*, *c*(*A*) = {*x* ∈ *L* : [*x*)∩*A* ≠ *∅*}.

**Proof**

The proof is trivial since [*x*) is the smallest *T*_*F*_-neighborhood of *x*.

Let *L* be a lattice implication algebra and let 2^*L*^ be the power set of *L*. A nonempty subset *I* of 2^*L*^ is called a power ideal of *L* if *I* satisfies the following: (1) *A*, *B* ∈ 2^*L*^ and *A*⊆*B* ∈ *I* imply *A* ∈ *I*; (2) *A*, *B* ∈ *I* implies *A* ∪ *B* ∈ *I*. The collection of all power ideals in 2^*L*^ is denoted by *I*(*L*), or briefly *I*. Note that *I*_*∅*_ = {*∅*} is the smallest power ideal and *I*_*L*_ = 2^*L*^ is the greatest power ideal. Moreover, if *I*, *J* ∈ *I*, then (1) *I*∩*J* ∈ *I*; (2) *I*∨*J* = {*I* ∪ *J* : *I* ∈ *I*, *J* ∈ *J*} ∈ *I*.

## 2. Local Functions and Power Idealization Filter Topologies

Let *L* be a lattice implication algebra, let *T*_*F*_ be the filter topology, and let *I* be a power ideal. An operator *on⁡  2^*L*^ is defined as follows:

(1) $$\begin{array}{c} A^{*}\left(I,T_{F}\right)=\left\{x\in L:\forall U\in N_{T_{F}}\left(x\right),A\cap U\notin I\right\} \end{array}$$

for every *A*⊆*L*.

The operator *is called the local function with respect to *T*_*F*_ and *I*. A* is called local function of *A*. We usually write A*(*I*) or A* instead of A*(*I*, *T*_*F*_).

Clearly, *x* ∈ A* if and only if [*x*)∩*A* ∉ *I*. Thus A* = {*x* ∈ *L* : [*x*)∩*A* ∉ *I*}. The following proposition gives some further details of A*.

**Proposition 2 .**

Let (*L*, *T*_*F*_) be the filter topological space and *I*, *J* ∈ *I*. Then

1. A*(*I*_*∅*_) = *c*(*A*) and A*(*I*_*L*_) = *∅*;
2. if *A*⊆*B*, then A*(*I*)⊆B*(*I*);
3. if *I*⊆*J*, then A*(*J*)⊆A*(*I*);
4. A*(*I*) = *c*(A*(*I*))⊆*c*(*A*);
5. (A*)*(*I*)⊆A*(*I*);
6. if *A*⊆*I*, then A*(*I*) = *∅*;
7. if *A* ∈ *T*_*F*_^*c*^, then A*(*I*)⊆*A*;
8. if *B* ∈ *I*, then (*A* ∪ *B*)*(*I*) = A*(*I*) = (*A*∖*B*)*(*I*);
9. (*A* ∪ A*(*I*))*(*I*) = A*(*I*);
10. (*A* ∪ *B*)*(*I*) = A*(*I*) ∪ B*(*I*);
11. A*(*I*)∖B*(*I*) = (*A*∖*B*)*(*I*)∖B*(*I*)⊆(*A*∖*B*)*(*I*);
12. if {1} ∉ *I*, then [*x*)*(*I*) = *L* for each *x* ∈ *L*;
13. if {1} ∉ *I* and 1 ∈ *A*⊆*L*, then [*A*)*(*I*) = [A*(*I*)) = *L*.

**Proof**

(1) By Proposition 1, *x* ∈ A*(*I*_*∅*_) if and only if [*x*)∩*A* ≠ *∅* if and only if *x* ∈ *c*(*A*). Thus A*(*I*_*∅*_) = *c*(*A*). Since [*x*)∩*A* ∈ 2^*L*^ = *I*_*L*_ for each *x* ∈ *L*, A*(*L*) = *∅*.

(2) Let *A*⊆*B* and *x* ∈ A*(*I*). Then [*x*)∩*A* ∉ *I*. Since *I* is a power ideal and [*x*)∩*A*⊆[*x*)∩*B*, [*x*)∩*B* ∉ *I* and so *x* ∈ B*(*I*). Thus A*(*I*)⊆B*(*I*).

(3) Let *I*⊆*J* and *x* ∈ A*(*I*). Then [*x*)∩*A* ∉ *J*. It follows that [*x*)∩*A* ∉ *I* and so *x* ∈ A*(*I*). Thus A*(*J*)⊆A*(*I*).

(4) If *x* ∉ *c*(*A*), then *x* ∈ *L*∖*c*(*A*) ∈ *T*_*F*_ and so [*x*)⊆*L*∖*c*(*A*). Thus [*x*)∩*A*⊆(*L*∖*c*(*A*))∩*A* = *∅* ∈ *I*. This implies *x* ∉ A*(*I*) and so A*(*I*)⊆*c*(*A*). Then *c*(A*(*I*))⊆*c*(*c*(*A*)) = *c*(*A*).

It is clear that A*(*I*)⊆*c*(A*(*I*)). Next, we prove *c*(A*(*I*))⊆A*(*I*).

Let *x* ∈ *c*(A*(*I*)). By Proposition 1, [*x*)∩A*(*I*) ≠ *∅*. Then there exists *y* ∈ [*x*)∩A*(*I*). By *y* ∈ A*(*I*), [*y*)∩*A* ∉ *I*. By *y* ∈ [*x*), [*y*)⊆[*x*). Thus [*x*)∩*A* ∉ *I* and so *x* ∈ A*(*I*). Therefore *c*(A*(*I*))⊆A*(*I*).

(5) By (4), (A*(*I*))*(*I*)⊆*c*(A*(*I*)) = A*(*I*).

(6) Since [*x*)∩*A*⊆*A* ∈ *I* for each *x* ∈ *L*, A*(*I*) = *∅*.

(7) Suppose that *x* ∈ A*(*I*)∖*A*. Then *x* ∈ *L*∖*A* ∈ *T*_*F*_. Thus [*x*)⊆*L*∖*A* and so [*x*)∩*A*⊆(*L*∖*A*) = *∅* ∈ *I*. Hence *x* ∉ A*(*I*) which is a contradiction. Therefore A*(*I*)⊆*A*.

(8) By (2), (*A*∖*B*)*(*I*)⊆A*(*I*)⊆(*A* ∪ *B*)*(*I*). Next, we prove the inverse inclusions.

If *x* ∉ (*A*∖*B*)*(*I*), then ([*x*)∩*A*)∖*B* = [*x*)∩(*A*∖*B*) ∈ *I*. Thus [*x*)∩*A*⊆*I* ∪ *B* ∈ *I* which follows from *I* is a power ideal. This implies *x* ∉ A*(*I*). Thus A*(*I*)⊆(*A*∖*B*)*(*I*) and so A*(*I*) = (*A*∖*B*)*(*I*).

If *x* ∉ A*(*I*), then [*x*)∩*A* ∈ *I*. Since *B* ∈ *I*,

(2) $$\begin{array}{c} \left[x\right)\cap \left(A\cup B\right)\subseteq \left(\left[x\right)\cap A\right)\cup B\in I. \end{array}$$

Thus *x* ∉ (*A* ∪ *B*)*(*I*). This implies (*A* ∪ *B*)*(*I*)⊆A*(*I*) and so (*A* ∪ *B*)*(*I*) = A*(*I*).

(9) Clearly, A*(*I*)⊆(*A* ∪ A*(*I*))*(*I*). Conversely, if *x* ∉ A*(*I*), then [*x*)∩*A* ∈ *I*. Let [*x*)∩*A* = *I*. Then *A*⊆*I* ∪ (*L*∖[*x*)). By (2), (7), (8), and [*x*) ∈ *T*_*F*_,

(3) $$\begin{array}{c} A^{*}\left(I\right)\subseteq {\left(I\cup \left(L\setminus \left[x\right)\right)\right)}^{*}\left(I\right)={\left(L\setminus \left[x\right)\right)}^{*}\left(I\right)\subseteq L\setminus \left[x\right). \end{array}$$

Thus *A* ∪ A*(*I*)⊆(*L*∖[*x*)) ∪ *A* and so

(4) [x)∩(A∪A∗(I))⊆((L∖[x))∪A)∩[x)=A∩[x)=I∈I.

This implies *x* ∉ (*A* ∪ A*(*I*))*(*I*) and so (*A* ∪ A*(*I*))*(*I*)⊆A*(*I*).

(10) A*(*I*) ∪ B*(*I*)⊆(*A* ∪ *B*)*(*I*) is clear. Conversely, if *x* ∉ A*(*I*) ∪ B*(*I*), then [*x*)∩*A*, [*x*)∩*B* ∈ *I*. Thus [*x*)∩(*A* ∪ *B*) = ([*x*)∩*A*)∪([*x*) ∪ *B*) ∈ *I*. This implies *x* ∉ (*A* ∪ *B*)*(*I*). Therefore (*A* ∪ *B*)*(*I*)⊆A*(*I*) ∪ B*(*I*).

(11) We firstly prove A*(*I*)∖B*(*I*)⊆(*A*∖*B*)*(*I*). Assume that *x* ∈ (A*(*I*)∖B*(*I*))∖(*A*∖*B*)*(*I*). Then [*x*)∩(*A*∖*B*) ∈ *I* and [*x*)∩*B* ∈ *I*. Thus

(5) [x)∩A⊆[x)∩((A∖B)∪B) =([x)∩(A∖B))∪([x)∩B)∈I.

This implies *x* ∉ A*(*I*) which is a contradiction. Thus A*(*I*)∖B*(*I*)⊆(*A*∖*B*)*(*I*) and so A*(*I*)∖B*(*I*)⊆(*A*∖*B*)*(*I*)∖B*(*I*). Finally, (*A*∖*B*)*(*I*)∖B*(*I*)⊆A*(*I*)∖B*(*I*) follows from (2). Therefore A*(*I*)∖B*(*I*) = (*A*∖*B*)*(*I*)∖B*(*I*).

(12) Since {1} ∉ *I*, 1 ∉ *I* for each *I* ∈ *I*. Assume that there exists *x* ∈ *L* such that [*x*) ≠ *L*. Let *y* ∉ *L*∖[*x*)*(*I*). Thus [*y*)∩[*x*) ∈ *I*. Since [*x*∨*y*) = [*y*)∩[*x*), 1 ∉ [*x*∨*y*) which is a contradiction. Therefore [*x*)*(*I*) = *L* for each *x* ∈ *L*.

(13) Assume that there exists *y* ∈ *L*∖A*(*I*). Then 1 ∈ [*y*)∩*A* ∈ *I* which is a contradiction. Thus A*(*I*) = *L* and [A*(*I*)) = *L*. Since 1 ∈ *A*, *L* = [1)*(*I*)⊆[*A*)*(*I*) follows from (12). Therefore [*A*)*(*I*) = *L*.

**Proposition 3 .**

Let (*L*, *T*_*F*_) be the filter topological space and *I* ∈ *I*. The operator *c*_I_* (briefly, c*) on 2^*L*^, defined by c*(*A*) = *A* ∪ A* for *A*⊆*L*, satisfies the following statements:

1. c*(*∅*) = *∅*; c*(*L*) = *L*,
2. c*(c*(*A*)) = c*(*A*)⊆*c*(*A*),
3. (c*(*A*))* = c*(A*) = A*,
4. c*(*A* ∪ *B*) = c*(*A*) ∪ c*(*B*),
5. *A* ∈ *T*_*F*_^*c*^ or *A* ∈ *I* implies c*(*A*) = *A*.

**Proof**

(1) c*(*∅*) = *∅* follows from ∅* = *∅*. c*(*L*) = *L* is clear.

(2) By (4) and (9) of Proposition 2,

(6) c∗(c∗(A))=(A∪A∗)∪(A∪A∗)∗=A∪A∗=c∗(A)⊆c(A).

(3) By (5) and (9) of Proposition 2,

(7) (c∗(A))∗=(c∗(A)∪(c∗(A))∗)∗=(A∪A∗∪(A∪A∗)∗)∗=(A∪A∗)∗=A∗

and c*(A*) = A* ∪ (A*)* = A*.

(4) By (10) of Proposition 2,

(8) c∗(A∪B)=(A∪B)∪(A∪B)∗=(A∪A∗)∪(B∪B∗)=c∗(A)∪c∗(B).

(5) The result follows from (6) and (7) of Proposition 2.

**Theorem 4 .**

Let (*L*, *T*_*F*_) be the filter topological space and *I* ∈ *I*. The operator c* stated in Proposition 3 is the closure operator of a new topology which is finer than *T*_*F*_ and the topology generated by *I* ^*c*^ (note that *I* ^*c*^ is not a topology since *∅* ∉ *I* ^*c*^ in general case). Such a topology is called a power idealization filter topology and often denoted by *T*_F_*(*I*, *T*_*F*_), *T*_F_*(*I*), or *T*_F_*.

**Proof**

Let *T*_F_* = {*A*⊆*L* : c*(*L*∖*A*) = *L*∖*A*}. We prove that *T*_F_* is a topology on *L*.

(1) *∅*, *L* ∈ *T*_F_* follows from (1) of Proposition 3.

(2) If *A*, *B* ∈ *T*_F_*, then c*(*L*∖*A*) = *L*∖*A* and c*(*L*∖*B*) = *L*∖*B*. Thus

(9) c∗(L∖(A∩B))=c∗((L∖A)∪(L∖B))=c∗(L∖A)∪c∗(L∖B)=L∖(A∩B).

This implies *A*∩*B* ∈ *T*_F_*.

(3) Let *A*_*t*_ ∈ *T*_F_* for *t* ∈ *T*, where *T* is an index set. Then c*(*L*∖*A*_*t*_) = *L*∖*A*_*t*_ and

(10) L∖(∪t∈T)=∩t∈T(L∖At)⊆c∗(∩t∈T(L∖At))=∩t∈T(L∖At)∪(∩t∈T(L∖At))∗⊆∩t∈T((L∖At)∪(L∖At)∗)=∩t∈Tc∗(L∖At)=∩t∈T(L∖At)=L∖(∪t∈TAt).

Therefore ∪_*t*∈*T*_ *A*_*t*_ ∈ *T*_F_*.

Finally, by (6) and (7) of Proposition 3, *T*_*F*_, *I* ^*c*^⊆*T*_F_*.

**Example 5 .**

**Figure 1:**
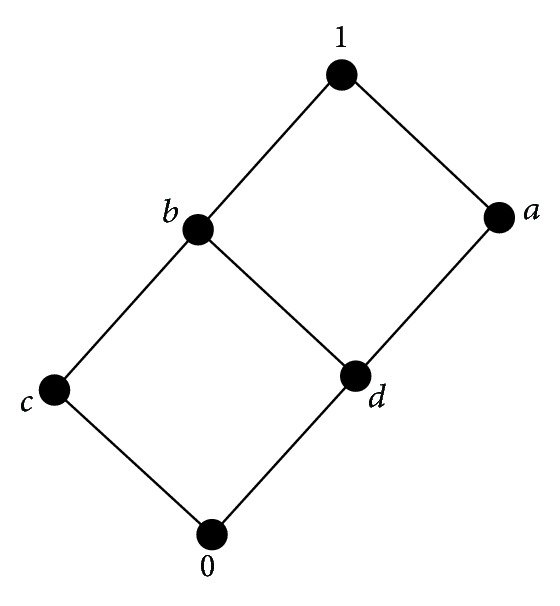
Hasse diagram of *L*_2_ = {0, *a*, *b*, *c*, *d*, 1}.

Let *L* = {0, *a*, *b*, *c*, *d*, 1}, 0′ = 1, *a*′ = *c*, *b*′ = *d*, *c*′ = *a*, *d*′ = *b*, 1′ = 0, and the implication operator → be defined by *a* → *b* = *a*′∨*b* for *a*, *b* ∈ *L*. Then (*L*, ∧, ∨,  ′, →) is the Hasse lattice implication algebra (Figure 1 and Table 1). Then

(11) $$\begin{array}{c} F\left(L\right)=\left\{\left\{1\right\},\left\{a,1\right\},\left\{b,c,1\right\},\left\{a,b,c,d,1\right\},L\right\},\\ T_{F(L)}=\left\{\emptyset ,\left\{1\right\},\left\{a,1\right\},\left\{b,c,1\right\},\left\{a,b,c,1\right\},\left\{a,b,c,d,1\right\},L\right\}. \end{array}$$

Let *I* = {*∅*, {0}, {*a*}, {0, *a*}}. Then *I* is a power ideal. It is easy to check that

(12) TF∗={∅,{1},{a,1},{b,c,1},{a,b,c,1},{b,c,d,1},  {0,b,c,d,1},{a,b,c,d,1},L}.

Clearly, *T*_*F*_⊆*T*_F_*.

**Proposition 6 .**

Let (*L*, *T*_*F*_) be the filter topological space and *I*, *J* ∈ *I*. Then

1. *T*_F_*(*I*_*∅*_) = *T*_*F*_ and *T*_F_*(*I*_*L*_) = 2^*L*^,
2. if *I*⊆*J*, then *T*_F_*(*I*)⊆*T*_F_*(*J*).

**Proof**

(1) By (1) of Proposition 2, A*(*I*_*∅*_) = *c*(*A*) and A*(*I*_*L*_) = *∅*. Thus *c*_*I*_∅__*(*A*) = *A* if and only if *c*(*A*) = *A*. Similarly, *c*_*I*_L__*(*A*) = *A* for each *A*⊆*L*. Therefore (1) holds.

(2) Let *I*⊆*J*. By (3) of Proposition 2, *c*_J_*(*A*)⊆*c*_I_*(*A*) for *A*⊆*L*. Thus if *A* ∈ *T*_F_*(*I*), then *A* ∈ *T*_F_*(*J*). Therefore *T*_F_*(*I*)⊆*T*_F_*(*J*).

Clearly, if *I* ∈ *I* satisfies *T*_*F*_^*c*^⊆*I*, then *L* ∈ *I* and so *I* = 2^*L*^ = *I*_*L*_. Thus by (1) of Proposition 3, *T*_F_* = *I* ^*c*^ = 2^*L*^. If *T*_*F*_^*c*^∖{*L*}⊆*I*, we have the following proposition.

**Proposition 7 .**

Let (*L*, *T*_*F*_) be the filter topological space. If *I* ∈ *I* satisfies *T*_*F*_^*c*^∖{*L*}⊆*I*, then *T*_F_* = *I* ^*c*^ ∪ {*∅*}.

**Proof**

*I* ^*c*^ ∪ {*∅*}⊆*T*_F_* follows from Theorem 4. Conversely, suppose that *T*_F_*⊈*I* ^*c*^ ∪ {*∅*}. Then there exists *B* ∈ *T*_F_*∖(*I* ^*c*^ ∪ {*∅*}) such that (*L*∖*B*)*⊆*L*∖*B* ≠ *L*. Let *y* ∈ (*L*∖*B*)∖(*L*∖*B*)*. Then *y* ∉ (*L*∖*B*)*. Thus [*y*)∩(*L*∖*B*) ∈ *I*. Put [*y*)∩(*L*∖*B*) = *I*. We have *L*∖*B*⊆*I* ∪ (*L*∖[*y*)). Since *T*_*F*_^*c*^∖{*L*}⊆*I*, *L*∖[*y*) ∈ *I*. Thus *L*∖*B*⊆*I* ∪ (*L*∖*B*) ∈ *I* and so *L*∖*B* ∈ *I*. Hence *B* ∈ *I* ^*c*^. It is a contradiction. Therefore *T*_F_*⊆*I* ^*c*^ ∪ {*∅*}.

**Lemma 8 .**

Let (*L*, *T*_*F*_) be the filter topological space and *I* ∈ *I*. If *A*⊆*L* satisfies *A*∩*I* = *∅* for each *I* ∈ *I*, then c*(*A*) = *c*(*A*).

**Proof**

c*(*A*)⊆*c*(*A*) is clear. Conversely, if *x* ∉ c*(*A*), then *x* ∉ *A* and *x* ∉ A*. Thus *I* = [*x*)∩*A* ∈ *I* and *A*⊆*I* ∪ (*L*∖[*x*)). Since *A*∩*I* = *∅*, *A*⊆*L*∖[*x*). Observe that *x* ∉ *L*∖[*x*) and *L*∖[*x*) is *T*_*F*_-closed. We have *x* ∉ *c*(*A*). Thus *c*(*A*)⊆c*(*A*). Therefore c*(*A*) = *c*(*A*).

**Proposition 9 .**

If *I* ∈ *T*_*F*_^*c*^, then *T*_F_* = *T*_*F*_.

**Proof**

It is clear that *T*_*F*_⊆*T*_F_*. Conversely, let *I*_*M*_ be the greatest element of *I* and *A*⊆*L*. Thus (*A*∖*I*_*M*_)∩*I* = *∅* for each *I* ∈ *I*. By Lemma 8, c*(*A*∖*I*_*M*_) = *c*(*A*∖*I*_*M*_). By (8) of Proposition 2, (*A*∖*I*_*M*_)* = A*. Now, notice that *A*∩*I*_*M*_ ∈ *I*⊆*T*_*F*_^*c*^ and thus *c*(*A*∩*I*_*M*_) = *A*∩*I*_*M*_. We have

(13) c(A)=c((A∖IM)∪(A∩IM))=c(A∖IM)∪c(A∩IM)=c∗(A∖IM)∪(A∩IM)=(A∖IM)∪(A∖IM)∗∪(A∩IM)=(A∖IM)∗∪A=A∗∪A=c∗(A).

This implies c* = *c*. Therefore *T*_F_* = *T*_*F*_.

**Lemma 10 .**

Let (*L*, *T*_*F*_) be the filter topological space and *I* ∈ *I*. If *I* ∈ *I* and *U* ∈ *T*_*F*_, then *U*∖*I* ∈ *T*_F_*.

**Proof**

Let *P* = *L*∖*U*. Then *P* ∈ *T*_*F*_^*c*^. By (7) and (8) of Proposition 2,

(14) $$\begin{array}{c} c^{*}\left(P\cup I\right)=\left(P\cup I\right)\cup {\left(P\cup I\right)}^{*}=\left(P\cup I\right)\cup P^{*}=P\cup I. \end{array}$$

Thus *L*∖(*P* ∪ *I*) = *U*∩(*L*∖*I*) = *U*∖*I* ∈ *T*_F_*.

**Theorem 11 .**

Let (*L*, *T*_*F*_) be the filter topological space and *I* ∈ *I*. Then

(15) $$\begin{array}{c} B_{T_{F}^{*}}=\left\{U\setminus I:U\in T_{F},I\in I\right\} \end{array}$$

is a base of *T*_F_*. Moreover,

(16) $$\begin{array}{c} B_{T_{F}^{*}}\left(x\right)=\left\{V\setminus I:V\in N_{T_{F}}\left(x\right),\;x\notin I\in I\right\} \end{array}$$

is a base of *N*_*T*_F_*_(*x*) for each *x* ∈ *L*, where *N*_*T*_F_*_(*x*) is the set of all *T*_F_*-neighborhoods of *x* in (*L*, *T*_F_*). Clearly, [*x*)∖*I*_*x*_ is the smallest *T*_F_*-neighborhoods of *x*, where *I*_*x*_ is the greatest element of *I* satisfying *x* ∉ *I*_*x*_.

**Proof**

By Lemma 10, *B*_*T*_F_*_⊆*T*_F_*. Let *B*⊆*L*. Then *B* ∈ *T*_F_*⇔*L*∖*B* is *T*_F_*-closed ⇔(*L*∖*B*)*⊆(*L*∖*B*)⇔*B*⊆*L*∖(*L*∖*B*)*. Thus *x* ∈ *B*⇒*x* ∉ (*L*∖*B*)*⇒ there exists *U* ∈ *N*_*T*_*F*__(*x*) such that (*L*∖*B*)∩*U* ∈ *I*. Let *I* = (*L*∖*B*)∩*U*. Then *L*∖*B*⊆*I* ∪ (*L*∖*U*) and

(17) $$\begin{array}{c} x\in U\setminus I=U\cap \left(L\setminus I\right)=L\setminus \left(I\cup \left(L\setminus U\right)\right)\subseteq B. \end{array}$$

Therefore *B*_*T*_F_*_ is a base of *T*_F_*.

Clearly, *B*_*T*_F_*_(*x*)⊆*N*_*T*_F_*_(*x*). Let *A* ∈ *N*_*T*_F_*_(*x*) and *y* ∈ *A*. Since *B*_*T*_F_*_ is a base of *T*_F_*, there are *U*, *V* ∈ *T*_*F*_ and *I*, *J* ∈ *I* such that *x* ∈ *U*∖*I*⊆*A* and *y* ∈ *V*∖*J*⊆*A*. We can assume *y* ∉ *I* and *x* ∉ *J* (otherwise, *I* and *J* can be replaced by *I*∖{*y*} and *J*∖{*x*}, resp.,). Then

(18) (U∖I)∪(V∖J)=(U∩(L∖I))∪(V∩(L∖J))=[(U∪V)∩((L∖I)∪(L∖J))] ∩[(U∪(L∖J))∩(V∪(L∖I))] ⊇((U∪V)∖(I∩J))∩(L∖(I∪J))=(U∪V)∖(I∪J).  

Clearly, *y* ∈ (*U* ∪ *V*)∖(*I* ∪ *J*) ∈ *B*_*T*_F_*_(*x*) and

(19) $$\begin{array}{c} \left(U\cup V\right)\setminus \left(I\cup J\right)\subseteq \left(U\setminus I\right)\cup \left(V\setminus J\right)\subseteq A. \end{array}$$

Therefore *B*_*T*_F_*_(*x*) is a base of *N*_*T*_F_*_(*x*).

Clearly, if *I*_*x*_ is the greatest element of *I* satisfying *x* ∉ *I*_*x*_, then [*x*)∖*I*_*x*_ ∈ *N*_*T*_F_*_(*x*) is the smallest *T*_F_*-neighborhoods of *x*.

Let (*L*, *τ*) be a topological space and *I* ∈ *I*. The topology that was generated by *B* = {*U*∖*I* : *U* ∈ *τ*, *I* ∈ *I*} is denoted by T*(*I*, *τ*) [5]. Clearly, T*(*I*, *T*_*F*_) = *T*_F_*(*I*).

**Lemma 12 .**

Let *ψ* = {*∅*, *L*} be the indiscrete topology on *L* and *I* ∈ *I*. Then T*(*I*, *ψ*) = {*∅*} ∪ *I* ^*c*^.

**Proof**

By *T* ^*c*^∖{*L*} = {*∅*} ∈ *I* and Proposition 7, the proof is obvious.

**Theorem 13 .**

Let (*L*, *τ*) be a topological space and *I* ∈ *I*. Then T*(*I*, *τ*) = *τ*∨T*(*I*, *ψ*), where *τ*∨T*(*I*, *ψ*) is the topology generated by the base {*U*∩*V* : *U* ∈ *τ*, *V* ∈ T*(*I*, *ψ*)}.

**Proof**

Clearly, *B*_T*_ = {*U*∖*I* : *U* ∈ *τ*, *I* ∈ *I*} is a base of T*(*I*, *τ*). Since *B*_T*_ = {*U*∩*V* : *U* ∈ *τ*, *V* ∈ T*(*I*, *ψ*)}, *B*_T*_ is also a base of *τ*∨T*(*I*, *ψ*). Therefore T*(*I*, *τ*) = *τ*∨T*(*I*, *ψ*).

**Corollary 14 .**

Let (*L*, *T*_*F*_) be the filter topological space and *I* ∈ *I*. Then *T*_F_* = *T*_*F*_∨T*(*I*, *ψ*).

**Corollary 15 .**

Let (*L*, *T*_*F*_) be the filter topological space and *I*, *J* ∈ *I*. Then

1. T*(*I*∨*J*, *ψ*) = T*(*I*, *ψ*)∨T*(*J*, *ψ*),
2. *T*_F_*(*I*∨*J*) = T*(*I*, *T*_F_*(*J*)) = T*(*J*, *T*_F_*(*I*)),
3. *T*_F_*(*I*∨*J*) = *T*_F_*(*I*)∨*T*_F_*(*J*),
4. T*(*I*, *T*_F_*(*I*)) = *T*_F_*(*I*).

**Proof**

(1) By (2) of Proposition 6, T*(*I*∨*J*)⊇T*(*I*)∨T*(*J*). Conversely, let *∅* ≠ *A* ∈ T*(*I*∨*J*). By Theorem 13, there exist *I* ∈ *I* and *J* ∈ *J* such that

(20) $$\begin{array}{c} A=L\setminus \left(I\cup I\right)=\left(L\setminus I\right)\cap \left(L\setminus J\right)\in T^{*}\left(I\right)\vee T^{*}\left(J\right). \end{array}$$

Thus T*(*I*∨*J*)⊆T*(*I*)∨T*(*J*).

(2) By (1), Theorem 13, and Corollary 14,

(21) TF∗(I∨J)=TF∨T∗(I∨J,ψ)=TF∨T∗(I,ψ)∨T∗(J,ψ)=TF∗(I)∨T∗(J,ψ)=T∗(TF∗(I),J).

Similarly, *T*_F_*(*I*∨*J*) = T*(*T*_F_*(*J*), *I*).

(3) By (1) and Theorem 13,

(22) TF∗(I∨J)=TF∨T∗(I∨J,ψ)=TF∨T∗(I,ψ)∨T∗(J,ψ)=TF∨T∗(I,ψ)∨TF∨T∗(J,ψ)=TF∗(I)∨TF∗(J).

Therefore *T*_F_*(*I*∨*J*) = *T*_F_*(*I*)∨*T*_F_*(*J*).

(4) Let *I* = *J*. Then the proof follows from (2).

**Theorem 16 .**

Let (*L*, *T*_*F*_) be the filter topological space, *I*, *J* ∈ *I* and *A*⊆*L*. Then

1. A*(*I*∩*J*, *T*_*F*_) = A*(*I*, *T*_*F*_) ∪ A*(*J*, *T*_*F*_),
2. A*(*I*∨*J*, *T*_*F*_) = A*(*I*, *T*_F_*(*J*))∩A*(*J*, *T*_F_*(*I*)).

**Proof**

(1) By (3) of Proposition 2, A*(*I*, *T*_*F*_) ∪ A*(*J*, *T*_*F*_)⊆A*(*I*∩*J*, *T*_*F*_). Conversely, *x* ∉ A*(*I*, *T*_*F*_) ∪ A*(*J*, *T*_*F*_). Then [*x*)∩*A* ∈ *I* and [*x*)∩*A* ∈ *J*. Let [*x*)∩*A* = *I* and [*x*)∩*A* = *J*. Then *A*⊆*I* ∪ (*L*∖[*x*)) and *A*⊆*J* ∪ (*L*∖[*x*)). Thus

(23) $$\begin{array}{c} A\subseteq \left(I\cup \left(L\setminus \left[x\right)\right)\right)\cap \left(J\cup \left(L\setminus \left[x\right)\right)\right)=\left(I\cap J\right)\cup \left(L\setminus \left[x\right)\right). \end{array}$$

Thus [*x*)∩*A*⊆*I*∩*J* which implies *x* ∉ A*(*I*∩*J*, *T*_*F*_). Therefore A*(*I*∩*J*, *T*_*F*_)⊆A*(*I*, *T*_*F*_) ∪ A*(*J*, *T*_*F*_).

(3) Let *x* ∉ A*(*I*∨*J*, *T*_*F*_). Then [*x*)∩*A* ∈ *I*∨*J*. Then there exist *I* ∈ *I* and *J* ∈ *J* such that [*x*)∩*A* = *I* ∪ *J*. We can assume *I*∩*J* = *∅*, (otherwise, *I* can be replaced by *I*∖(*I*∩*J*)). Thus *x* ∉ *I* or *x* ∉ *J*, (otherwise, *x* ∈ *I*∩*J* which is a contradiction). Now, we take *x* ∉ *I* for example. Then

(24) $$\begin{array}{c} \left(\left[x\right)\setminus I\right)\cap A=\left[x\right)\cap A\cap \left(L\setminus I\right)=J. \end{array}$$

Since [*x*) ∈ *T*_*F*_ and *x* ∈ [*x*)∖*I*, [*x*)∖*I* ∈ *B*_*T*_F_*(*I*)_. Thus *x* ∉ A*(*J*, *T*_F_*(*I*)) and so *x* ∉ A*(*J*, *T*_F_*(*I*))∩A*(*I*, *T*_F_*(*J*)). Hence

(25) $$\begin{array}{c} A^{*}\left(J,T_{F}^{*}\left(I\right)\right)\cap A^{*}\left(I,T_{F}^{*}\left(J\right)\right)\subseteq A^{*}\left(I\vee J,T_{F}\right). \end{array}$$

Conversely, let *x* ∉ A*(*T*_F_*(*I*), *J*). Then there exists *I* ∈ *I* such that ([*x*)∖*I*)∩*A* ∈ *J*. Let ([*x*)∖*I*)∩*A* = *J*. Then [*x*)∩*A* = *I* ∪ *J* which implies *x* ∉ A*(*I*∨*J*, *T*_*F*_). Similarly, if *x* ∉ A*(*T*_F_*(*J*), *I*), then *x* ∉ A*(*I*∨*J*, *T*_*F*_). Therefore A*(*I*∨*J*, *T*_*F*_)⊆A*(*J*, *T*_F_*(*I*))∩A*(*I*, *T*_F_*(*J*)).

**Corollary 17 .**

Consider A*(*I*, *T*_*F*_) = A*(*I*, *T*_F_*(*I*)).

**Proof**

Let *I* = *J*. The proof follows from (2) of Theorem 16.

**Corollary 18 .**

Consider *T*_F_*(*I*∩*J*) = *T*_F_*(*I*)∩*T*_F_*(*J*).

**Proof**

By (2) of Proposition 6, *T*_F_*(*I*∩*J*)⊆*T*_F_*(*I*)∩*T*_F_*(*J*). Conversely, if *A* ∉ *T*_F_*(*I*∩*J*), then

(26) (L∖A)∗(I,TF)∪(L∖A)∗(J,TF) =(L∖A)∗(I∩J,TF)⊈(L∖A).

Thus (*L*∖*A*)*(*I*, *T*_*F*_)⊈(*L*∖*A*) or (*L*∖*A*)*(*J*, *T*_*F*_)⊈(*L*∖*A*). Thus *A* ∉ *T*_F_*(*I*) or *A* ∉ *T*_F_*(*J*). Therefore *T*_F_*(*I*)∩*T*_F_*(*J*)⊆*T*_F_*(*I*∩*J*).

## 3. Power Idealization Filter Topological Quotient Spaces

Let (*L*_1_, ∧, ∨,  ′, →_1_, 0_1_, 1_1_) and (*L*_2_, ∧, ∨,  ′, →_2_, 0_2_, 1_2_) be two lattice implication algebras. A mapping *f* from *L*_1_ to *L*_2_ is called lattice implication homomorphism, if *f*(*x*→_1_ *y*) = *f*(*x*)→_2_ *f*(*y*) for any *x*, *y* ∈ *L*_1_. The set of all lattice implication homomorphisms from *L*_1_ to *L*_2_ is denoted by hom(*L*_1_, *L*_2_).

Let *f* ∈ hom(*L*_1_, *L*_2_). Then, clearly,

(27) $$\begin{array}{c} T_{l}\left(T_{F(L_{2})},f\right)=\left\{f^{-1}\left(U\right):U\in T_{F(L_{2})}\right\},\\ T_{r}\left(T_{F(L_{1})},f\right)=\left\{f\left(U\right):U\subseteq L_{2},f^{-1}\left(U\right)\in T_{F(L_{1})}\right\} \end{array}$$

are topologies [4].

**Lemma 19 .**

Let *L*_1_ and *L*_2_ be two implication algebras and let *f* ∈ hom(*F*_1_, *f*_2_) and *I* ∈ 2^*L*_1_^, *J* ∈ 2^*L*_2_^ be power ideals.

1. If *f* is injective, then *f* ^−1^(*J*) = {*f* ^−1^(*J*) : *J* ∈ *J*} ∈ *I*(*L*_1_).
2. If *f* is surjective, then *f*(*I*) = {*f*(*I*) : *I* ∈ *I*} ∈ *I*(*L*_2_).

**Proof**

(1) Since *∅* ∈ *J*, then *∅* = *f* ^−1^(*∅*) ∈ *f* ^−1^(*J*).

If *I*_2_ ∈ *f* ^−1^(*J*) and *I*_1_⊆*I*_2_, then there exist *J*_2_ ∈ *J* such that *I*_2_ = *f* ^−1^(*J*_2_). Thus *f*(*I*_1_)⊆*f*(*I*_2_) = *J*_2_ and *f*(*I*_1_) ∈ *J*. Since *f* is injective, *I*_1_ = *f* ^−1^(*f*(*I*_1_)) ∈ *f* ^−1^(*J*).

If *I*_1_, *I*_2_ ∈ *f* ^−1^(*J*), then there exist *J*_1_, *J*_2_ ∈ *J* such that *I*_1_ = *f* ^−1^(*J*_1_) and *I*_2_ = *f* ^−1^(*J*_2_). One has *I*_1_ ∪ *I*_2_ = *f* ^−1^(*J*_1_) ∪ *f* ^−1^(*J*_2_) = *f* ^−1^(*J*_1_ ∪ *J*_2_). Since *J*_1_ ∪ *J*_2_ ∈ *J*, *I*_1_ ∪ *I*_2_ ∈ *f* ^−1^(*J*). Therefore *f* ^−1^(*J*) is a power ideal.

(2) By *∅* ∈ *I*, *∅* = *f*(*∅*) ∈ *f*(*I*).

If *J*_2_ ∈ *f*(*J*) and *J*_1_⊆*J*_2_, then there exists *I*_2_ ∈ *I* such that *J*_2_ = *f*(*I*_2_). Let *I*_1_ = *f* ^−1^(*J*_1_). Then *I*_1_⊆*I*_2_ and *I*_1_ ∈ *I*. Since *f* is surjective, *J*_1_ = *f*(*I*_1_) ∈ *f*(*I*).

If *J*_1_, *J*_2_ ∈ *f*(*J*), then there exist *I*_1_, *I*_2_ ∈ *I* such that *J*_1_ = *f*(*I*_1_) and *J*_2_ = *f*(*I*_2_). Thus *I*_1_ ∪ *I*_2_ ∈ *I*. Hence *J*_1_ ∪ *J*_2_ = *f*(*I*_1_) ∪ *f*(*I*_2_) = *f*(*I*_1_ ∪ *I*_2_) ∈ *f*(*I*). Therefore *f*(*I*) is a power ideal.

**Lemma 20 .**

Let *L*_1_ and *L*_2_ be two implication algebras and *f* ∈ hom(*F*_1_, *F*_2_). Then

1. if *f* is injective, then for each *x* ∈ *L*_1_, *f* ^−1^([*f*(*x*))) ∈ *T*_*l*_(*T*_*F*(*L*_2_)_, *f*) is the smallest *T*_*l*_-neighborhood of *x*;
2. if *f* is bijective, then for each *y* ∈ *L*_2_, *f*([*f* ^−1^(*y*))) ∈ *T*_*r*_(*T*_*F*(*L*_1_)_, *f*) is the smallest *T*_*r*_-neighborhood of *y*.

**Proof**

(1) Clearly, [*f*(*x*)) is the smallest *T*_*F*(*L*_2_)_-neighborhood of *f*(*x*). Then *x* ∈ *f* ^−1^([*f*(*x*))) ∈ *T*_*l*_(*T*_*F*(*L*_2_)_, *f*). Let *x* ∈ *V* ∈ *T*_*l*_(*T*_*F*(*L*_2_)_, *f*). Then *f*(*x*) ∈ *f*(*V*) ∈ *T*_*F*(*L*_2_)_ and [*f*(*x*))⊆*f*(*V*). Thus *f* ^−1^([*f*(*x*)))⊆*f* ^−1^(*f*(*V*)) = *V*. Therefore *f* ^−1^([*f*(*x*))) is the smallest one.

(2) Clearly, [*f* ^−1^(*y*)) is the smallest *T*_*F*(*L*_1_)_-neighborhood of *f* ^−1^(*y*). Thus *y* ∈ *f*([*f* ^−1^(*y*))) ∈ *T*_*r*_(*T*_*F*(*L*_1_)_, *f*). Now, let *y* ∈ *U* ∈ *T*_*r*_(*T*_*F*(*L*_1_)_, *f*). Then *f* ^−1^(*y*) ∈ *f* ^−1^(*U*) ∈ *T*_*F*(*L*_1_)_. Thus [*f* ^−1^(*y*))⊆*f* ^−1^(*U*) and so *f*([*f* ^−1^(*y*)))⊆*f*(*f* ^−1^(*U*)) = *U*. Therefore (2) holds.

**Lemma 21 .**

Let *L*_1_ and *L*_2_ be two implication algebras and *f* ∈ hom(*F*_1_, *F*_2_).

1. If *f* is injective and *J* ∈ *I*(*L*_2_), then for each *x* ∈ *L*_1_, [*f*(*x*))∖*J*_*M*_ ∈ *T*_F_*(*J*, *T*_*F*(*L*_2_)_) is the smallest neighborhood of *f*(*x*), where *J*_*M*_ is the greatest element of *J* satisfying *f*(*x*) ∉ *J*_*M*_.
2. If *f* is bijective and *I* ∈ *I*(*L*_1_), then for each *y* ∈ *L*_2_, [*f* ^−1^(*y*))∖*I*_*M*_ ∈ *T*_F_*(*I*, *T*_*F*(*L*_1_)_) is the smallest neighborhood of *f* ^−1^(*y*), where *I*_*M*_ is the greatest element of *I* satisfying *f* ^−1^(*y*) ∉ *I*_*M*_.

**Theorem 22 .**

Let *L*_1_ and *L*_2_ be two implication algebras and *f* ∈ hom(*F*_1_, *F*_2_).

(1) If *f* is injective and *J* ∈ *I*(*L*_2_), then
  (28) $$\begin{array}{c} T^{*}\left(f^{-1}\left(J\right),T_{l}\left(T_{F(L_{2})},f\right)\right)=T_{l}\left(T_{F}^{*}\left(J,T_{F(L_{2})}\right),f\right). \end{array}$$
(2) If *f* is bijective and *I* ∈ *I*(*L*_1_), then
  (29) $$\begin{array}{c} T^{*}\left(f\left(I\right),T_{r}\left(T_{F(L_{1})},f\right)\right)=T_{r}\left(T_{F}^{*}\left(I,T_{F(L_{1})}\right),f\right). \end{array}$$

**Proof**

(1) Let *c*_2_* and *c*_*l*_ be the closure operators of the left side and the right side of the equation. We only need to prove *c*_1_* = *c*_*l*_.

Let *A*⊆*L*_1_ and *x* ∉ *c*_1_*(*A*). Then *x* ∉ *A* and *x* ∉ A*(*f* ^−1^(*J*), *T*_*l*_(*T*_*F*(*L*_2_)_, *f*)). By (1) of Lemma 21, *f* ^−1^([*f*(*x*)))∩*A* ∈ *f* ^−1^(*J*). Thus there exists *J* ∈ *J* such that *f* ^−1^([*f*(*x*)))∩*A* = *f* ^−1^(*J*). Since *x* ∉ *A* and *f* is injective, *f*(*x*) ∉ *J*. Let *J*_*M*_ ∈ *J* be the greatest one satisfying *f*(*x*) ∉ *J*_*M*_. Then *f* ^−1^([*f*(*x*)))∩*A*⊆*f* ^−1^(*J*_*M*_). Thus

(30) ∅=f−1([f(x)))∩A∩(L1∖f−1(JM))=f−1([f(x)))∩f−1(L2∖JM)∩A=f−1([f(x))∩(L2∖JM))∩A=f−1([f(x))∖JM)∩A.

By Lemma 21 and Proposition 1, *x* ∉ *c*_*l*_(*A*). Therefore *c*_*l*_(*A*)⊆*c*_1_*(*A*).

Conversely, let *y* ∉ *c*_*l*_(*A*). By Proposition 1,

(31) ∅=f−1([f(x))∖JM)∩A=f−1([f(x)))∩A∩(L1∖f−1(JM)).

Thus *x* ∉ *A*, *f* ^−1^([*f*(*x*)))∩*A*⊆*f* ^−1^(*J*_*M*_) and [*f*(*x*))∩*f*(*A*)⊆*J*_*M*_. Then *J*_1_ = [*f*(*x*))∩*f*(*A*) ∈ *J*. Since *f* is injective, *f* ^−1^([*f*(*x*)))∩*A* = *f* ^−1^(*J*_1_). By (1) of Lemma 20, *x* ∉ A*(*f* ^−1^(*J*), *T*_*l*_(*T*_*F*(*L*_2_)_, *f*)). Therefore *x* ∉ *c*_1_*(*A*) and *c*_1_*(*A*)⊆*c*_*l*_(*A*).

(2) Let *c*_2_* and *c*_*r*_ be the closure operators of the left side and the right side of the equation. We only need to prove *c*_2_* = *c*_*r*_.

Let *y* ∉ *c*_2_*(*A*). Then *y* ∉ *A* and *y* ∉ A*((*f*(*I*), *T*_*r*_(*T*_*F*(*L*_1_)_, *f*))). By (2) of Lemma 20, *f*([*f* ^−1^(*y*)))∩*A* ∈ *f*(*I*). Thus there exists *I* ∈ *I* such that *f*([*f* ^−1^(*y*)))∩*A* = *f*(*I*). Since *f* is bijective, [*f* ^−1^(*y*))∩*f* ^−1^(*A*) = *I*. By *y* ∉ *A*, *f* ^−1^(*y*) ∉ *f* ^−1^(*A*) and so *f* ^−1^(*y*) ∉ *I*. Since *I*_*M*_ ∈ *I* is the greatest element of *I* satisfying *f* ^−1^(*y*) ∉ *I*_*M*_, [*f* ^−1^(*y*))∩*f* ^−1^(*A*)⊆*I*_*M*_ and [*f* ^−1^(*y*))∩(*L*_1_∖*I*_*M*_)∩*f* ^−1^(*A*) = *∅*. By *f* being bijective again, we have

(32) ∅=f(∅)=f([f−1(y)))∩(L2∖f(IM))∩A=f([f−1(y))∖IM)∩A.

By

(33) f−1(y)∈[f−1(y))∖IM =f−1(f([f−1(y))∖IM))∈TF∗(I,TF(L1)),

*y* ∉ *c*_*r*_(*A*). Hence *c*_2_*(*A*)⊆*c*_*r*_(*A*).

Conversely, let *z* ∉ *c*_*r*_(*A*). Since *I*_*M*_ is the greatest element of *I* satisfying *f* ^−1^(*z*) ∉ *I*_*M*_, by (2) of Lemma 21, [*f* ^−1^(*z*))∖*I*_*M*_ ∈ *T*_F_*(*I*, *T*_*F*(*L*_1_)_) is the smallest neighborhood of *f* ^−1^(*z*). Thus *f*([*f* ^−1^(*z*))∖*I*_*M*_)∩*A* = *∅*. Since *f* is bijective,

(34) ∅=f−1(∅)=([f−1(z))∖IM)∩f−1(A)=[f−1(z))∩(L1∖IM)∩f−1(A).

This implies [*f* ^−1^(*z*))∩*f* ^−1^(*A*)⊆*I*_*M*_ and [*f* ^−1^(*z*))∩*f* ^−1^(*A*) ∈ *I*. Thus *f*([*f* ^−1^(*y*)))∩*A* ∈ *f*(*I*) which implies *y* ∈ A*((*f*(*I*), *T*_*r*_(*T*_*F*(*L*_1_)_, *f*))). Since *z* ∉ *c*_*r*_(*A*), *z* ∉ *A*. Therefore *z* ∉ *c*_2_*(*A*) and so *c*_2_*(*A*)⊆*c*_*r*_(*A*).

Generally, if *f* ∈ hom(*L*_1_, *L*_2_) is surjective but not bijective, then (2) of Theorem 22 fails.

**Example 23 .**

**Figure 2:**
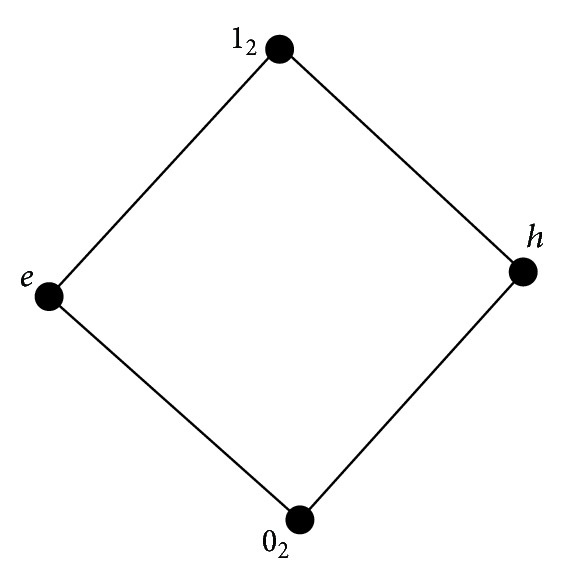
Hasse diagram of *L*_2_ = {0_2_, *e*, *h*, 1_2_}.

Let *L*_1_ = {0_1_, *a*, *b*, *c*, *d*, 1_1_} be the Hasse lattice implication algebra of Example 5. Let *L*_2_ = {0_2_, *e*, *h*, 1_2_} and 0_2_′ = 1_2_, *e*′ = *h*, *h*′ = *e*, and 1_2_′ = 0_2_. The Hasse diagram and the implication operator of *L*_2_ are shown by Figure 2 and Table 2. Then it is clear that

(35) TF(L1)∗={∅,{11},{a,11},{b,c,11},{a,b,c,11},  {b,c,d,11},{0,b,c,d,11},{a,b,c,d,11},L},TF(L2)∗={∅,{e,12},{h,12},{e,h,12},{12},L2}.

A mapping *f* from *L*_1_ to *L*_2_ is defined as

(36) $$\begin{array}{c} f\left(0_{1}\right)=0_{2},\;\;f\left(1_{1}\right)=1_{2},\;\;f\left(a\right)=f\left(d\right)=e,\\ f\left(b\right)=f\left(c\right)=h. \end{array}$$

It easy to check *f* ∈ hom(*L*_1_, *L*_2_) and *f* is surjective. Let *I* = {*∅*, {*c*}}. Then *I* ∈ *I*(*L*_1_) and *f*(*I*) = {*∅*, {*h*}} ∈ *I*(*L*_2_).

Since {*h*} ∈ *f*(*I*), by Theorem 4,

(37) $$\begin{array}{c} \left\{0_{2},e,1_{2}\right\}=L_{2}\setminus \left\{h\right\}\in T^{*}\left(f\left(I\right),T_{r}\left(T_{F(L_{1})},f\right)\right). \end{array}$$

Observe that {*b*, *c*}*(*I*, *T*_*F*(*L*_1_)_) = {0_1_, *b*, *c*, *d*}⊈{*b*, *c*}. We have

(38) $$\begin{array}{c} \left\{0,a,d,1\right\}=L_{1}\setminus \left\{b,c\right\}\notin T_{F}^{*}\left(I,T_{F(L_{1})}\right). \end{array}$$

Moreover, by *f* ^−1^({0_2_, *e*, 1_2_}) = {0_2_, *a*, *d*, 1_2_}, {0_2_, *e*, 1_2_} ∉ *T*_*r*_(*T*_F_*(*I*, *T*_*F*(*L*_1_)_), *f*).

In fact, we have

(39) $$\begin{array}{c} T_{r}\left(T_{F}^{*}\left(I,T_{F(L_{1})}\right),f\right)=\left\{\emptyset ,\left\{1_{2}\right\},L_{2}\right\},\\ T^{*}\left(f\left(I\right),T_{r}\left(T_{F(L_{1})},f\right)\right)=\left\{\emptyset ,\left\{1_{2}\right\},\left\{0_{2},e,1_{2}\right\},L_{2}\right\}. \end{array}$$

Therefore *T*_*r*_(*T*_F_*(*I*, *T*_*F*(*L*_1_)_), *f*) ≠ T*(*f*(*I*), *T*_*r*_(*T*_*F*(*L*_1_)_, *f*)).

**Corollary 24 .**

Let *L*_1_ and *L*_2_ be two implication algebras and let *f* ∈ hom(*F*_1_, *f*_2_) be bijective.

(1) If *J* ∈ *I*(*L*_2_), then
  (40) $$\begin{array}{c} T_{r}\left(T^{*}\left(f^{-1}\left(J\right),T_{l}\left(T_{F(L_{2})},f\right)\right),f\right)=T^{*}\left(J,T_{F(L_{2})}\right). \end{array}$$
(2) If *I* ∈ *I*(*L*_1_), then
  (41) $$\begin{array}{c} T_{l}\left(T^{*}\left(f\left(I\right),T_{r}\left(T_{F(L_{1})},f\right)\right),f\right)=T^{*}\left(I,T_{F(L_{1})}\right). \end{array}$$

**Proof**

The proof follows from Theorem 22.

**Corollary 25 .**

Let *L*_1_ and *L*_2_ be two implication algebras and *f* ∈ hom(*f*_1_, *f*_2_).

(1) If *f* is injective and *J*_1_, *J*_2_ ∈ *I*(*L*_2_), then
  (42) Tl(TF∗(J1∨J2,TF(L2)),f) =Tl(TF∗(J1,TF(L2)),f)∨Tl(TF∗(J2,TF(L2)),f).
(2) If *f* is bijective and *I*_1_, *I*_2_ ∈ *I*(*L*_1_), then
  (43) Tr(TF∗(I1∨I2,TF(L1)),f) =Tr(TF∗(I1,TF(L1)),f)∨Tr(TF∗(I2,TF(L1)),f).

**Proof**

(1) Clearly, *f* ^−1^(*J*_1_∨*J*_2_) = *f* ^−1^(*J*_1_)∨*f* ^−1^(*J*_2_). By (3) of Corollary 15 and Theorem 22,

(44) Tl(TF∗(J1∨J2,TF(L2)),f) =T∗(f−1(J1∨J2),Tl(TF(L2),f)) =T∗(f−1(J1),Tl(TF(L2),f))  ∨T∗(f−1(J2),Tl(TF(L2),f)) =Tl(TF∗(J1,TF(L2)),f)∨Tl(TF∗(J2,TF(L2)),f).

(2) Is similar to (1).

**Corollary 26 .**

Let *L*_1_ and *L*_2_ be two implication algebras and let *f* ∈ hom(*F*_1_, *f*_2_) be bijective. If *J* ∈ *I*(*L*_2_) and *I* ∈ *I*(*L*_1_), then

(45) T∗(f−1(J),Tl(TF∗(J,TF(L2)),f)) =T∗(f−1(J),Tl(TF(L2),f)),T∗(f(I),Tr(TF∗(I,TF(L1)),f)) =T∗(f(I),Tr(TF(L1),f)).

**Proof**

The proof follows from (4) of Corollary 15 and Theorem 22.

## Figures and Tables

**Table 1:** The implication operator of *L* = {0, *a*, *b*, *c*, *d*, 1}.

→	0	*a*	*b*	*c*	*d*	1
0	1	1	1	1	1	1
*a*	*c*	1	*b*	*c*	*b*	1
*b*	*d*	*a*	1	*b*	*a*	1
*c*	*a*	*a*	1	1	*a*	1
*d*	*b*	1	1	*b*	1	1
1	0	*a*	*b*	*c*	*d*	1

**Table 2:** The implication operator of *L*_2_ = {0_2_, *e*, *h*, 1_2_}.

→	0_2_	*e*	*h*	1_2_
0_2_	1	1_2_	1_2_	1_2_
*e*	*h*	1_2_	*h*	1_2_
*h*	*e*	*e*	1_2_	1_2_
1_2_	0_2_	*e*	*h*	1_2_

## References

[1] Lukasiewicz J (1920). On 3-valued logic. *Ruch Filozoficzny*.

[2] Xu Y (1993). Lattice implication algebra. *Journal of Southwest Jiaotong University*.

[3] Xu Y, Qin KY (1993). On filters of lattice implication algebras. *The Journal of Fuzzy Mathematics*.

[4] Xu Y, Ruan D, Qin K, Liu J (2003). *Lattice-Valued Logic*.

[5] Jankovic D, Hamlett TR (1990). New topologies from old via ideals. *The American Mathematical Monthly*.

